# A bifunctional lead–iron oxyfluoride, PbFeO_2_F, that functions as a visible-light-responsive photoanode and an electrocatalyst for water oxidation†

**DOI:** 10.1039/d1ra04793k

**Published:** 2021-07-23

**Authors:** Ryusuke Mizuochi, Kazunari Izumi, Yoshiyuki Inaguma, Kazuhiko Maeda

**Affiliations:** Department of Chemistry, School of Science, Tokyo Institute of Technology 2-12-1-NE-2 Ookayama Meguro-ku Tokyo 152-8550 Japan maedak@chem.titech.ac.jp; Department of Chemistry, Faculty of Science, Gakushuin University 1-5-1 Mejiro Toshima-ku Tokyo 171-8588 Japan

## Abstract

The oxyfluoride PbFeO_2_F was investigated as a photoanode material and as an electrocatalyst for water oxidation. PbFeO_2_F powder, which was synthesized by a high-pressure method and had an estimated bandgap of 2.1 eV, was deposited onto a fluorine-doped tin oxide (FTO) substrate. Mott–Schottky plot measurements for the PbFeO_2_F/FTO electrode showed n-type semiconductivity of PbFeO_2_F, with a flat-band potential of +0.53 ± 0.05 V *vs.* reversible hydrogen electrode (RHE). The PbFeO_2_F/FTO electrode, which was modified with a conductive TiO_2_ layer and a cobalt phosphate water-oxidation cocatalyst, showed a clear anodic photocurrent in aqueous K_3_PO_4_ solution under visible-light irradiation (*λ* < 600 nm). The PbFeO_2_F/FTO electrode without any modification functioned as a stable water-oxidation electrocatalyst to form O_2_ with a faradaic efficiency of close to unity. This study demonstrates that PbFeO_2_F is a bifunctional material, serving as a water-oxidation photoanode under a wide range of visible-light wavelengths and as an electrocatalyst that operates at a relatively low overpotential for water oxidation.

## Abstract 2

The PbFeO_2_F serves as a bifunctional material for a water-oxidation photoanode workable under a wide range of visible light and a water-oxidation electrocatalyst operatable at a relatively low overpotential.

## Graphical abstract

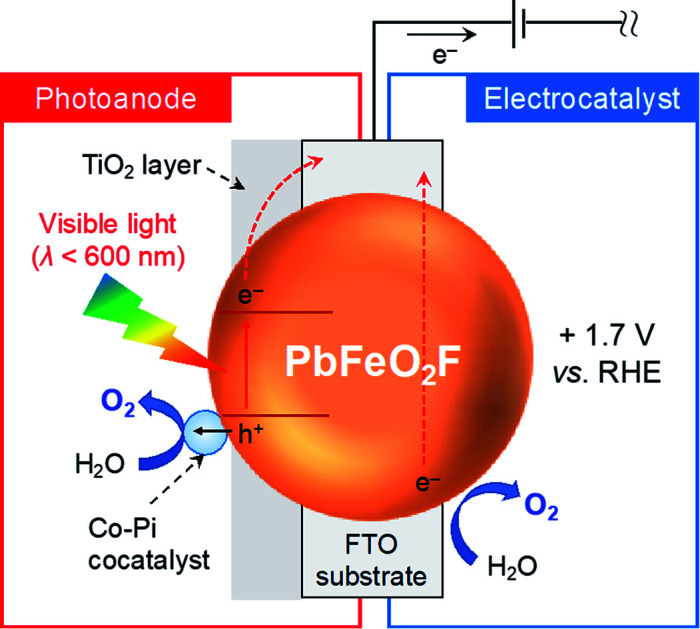

## Introduction

Hydrogen is expected to be used as a renewable energy carrier. Water splitting using semiconductor photoelectrodes or photocatalysts has attracted attention as a method of generating clean hydrogen using solar energy.^1–6^ Titanium-based metal oxides (*e.g.*, TiO_2_ (ref. 7) and SrTiO_3_ (ref. 8)) have been developed as stable photoanode materials for solar water oxidation but are not capable of efficiently utilizing visible light, which represents the majority of solar energy, because of their wide bandgaps (>3 eV). By contrast, visible-light-responsive metal oxides (*e.g.*, α-Fe_2_O_3_ (ref. 9–11) and BiVO_4_ (ref. 12–14)) unavoidably require an additional electrochemical (or external) bias for operation because their conduction-band minimum (CBM) is more positive than the H^+^/H_2_ reduction potential [0 V *vs.* standard hydrogen electrode (SHE) at pH 0].

Compared with the aforementioned oxide materials, mixed-anion compounds such as oxynitrides and oxysulfides have relatively small bandgaps and negative CBM potentials, making them good candidate photoanode materials for water oxidation under visible light.^3,15–17^ Some of them (*e.g.*, TaON^18,19^) theoretically enable water splitting to be driven under visible light without requiring an external bias because the CBM and the valence-band maximum (VBM) straddle the water reduction/oxidation potentials. However, the N 2p orbitals that constitute the VBM of oxynitrides are less stable than the O 2p orbitals, undergoing self-oxidation by holes generated during visible-light irradiation. This self-oxidation occurs with oxysulfides, in which the VBM is formed by S 3p orbitals. Thus, although the high-energy p-orbitals of anions are essential for providing small bandgaps, they are the main factor preventing more stable water oxidation by mixed-anion compounds.

Recently, the oxyfluoride Pb_2_Ti_2_O_5.4_F_1.2_, which is a mixed-anion compound, has been reported as a visible-light-absorbing photocatalyst with a narrow bandgap (∼2.4 eV) and n-type semiconductivity.^20–22^ Pb_2_Ti_2_O_5.4_F_1.2_ has a CBM at approximately −1.0 ± 0.1 V *vs.* SHE, which is sufficiently more negative than the water reduction potential.^21^ Thus, this oxyfluoride can drive standalone visible-light water splitting. Moreover, both the F 2p and O 2p orbitals are essentially stable toward self-oxidation by holes generated during visible-light irradiation of the oxyfluoride. Indeed, a Pb_2_Ti_2_O_5.4_F_1.2_ photoanode with visible-light responsivity and a relatively negative photocurrent onset potential has been reported.^23^ However, the literature includes only one example of a visible-light-responsive oxyfluoride photoelectrode (*i.e.*, Pb_2_Ti_2_O_5.4_F_1.2_) that can utilize a limited portion of visible light at wavelengths as long as 500 nm. Therefore, exploration of a new oxyfluoride photoelectrode that can absorb a greater range of visible-light wavelengths is important for enabling the design of visible-light-responsive photoelectrode materials.

Fe(iii)-containing materials are potentially useful as not only semiconductor photoanodes^9,11^ but also catalysts for water oxidation.^24–27^ The use of earth-abundant elements such as Fe is important for the development of water-oxidation photoanodes and/or catalysts not based on expensive metals, even if the performance of these materials initially found is moderate. Recently, oxyfluorides Co_3_Sb_4_O_6_F_6_,^28^ NiFe_2_F_4.4_O_1.8_,^29^ and CoFe_2_F_6.6_O_0.7_ (ref. 29) have been reported as electrocatalysts for water oxidation.

**Fig. 1:**
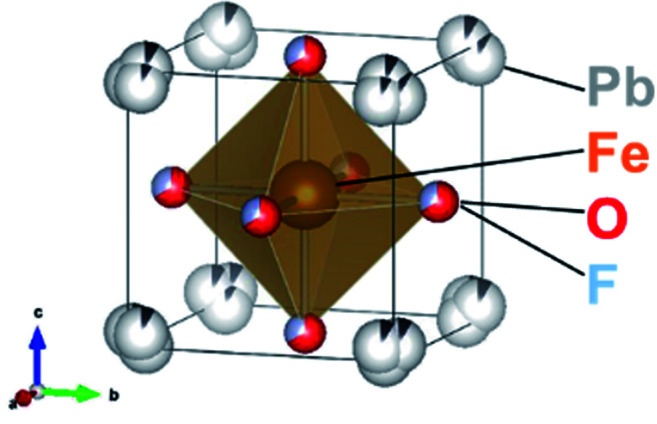
Crystal structure of PbFeO_2_F.

In the present work, the oxyfluoride PbFeO_2_F is examined as an electrode material for water splitting with and without irradiation. PbFeO_2_F, which can be synthesized by a high-pressure method,^30^ is an anion-disordered cubic perovskite with space group *Pm*3̄*m* (Fig. 1, drawn by the VESTA program^31^). PbFeO_2_F has been reported to exhibit antiferromagnetic behavior.^32^ It has also been reported to exhibit a yellow colour and is therefore expected to function as a photoelectrode material under visible light. In addition, the fact that PbFeO_2_F contains iron, an element that may provide active sites for water oxidation, suggests another functionality of electrocatalyst. Herein, we report that PbFeO_2_F can indeed function as both a semiconductor photoanode and electrocatalyst for water oxidation.

## Experimental

### Synthesis of PbFeO_2_F

PbFeO_2_F powder was synthesized *via* solid-state reaction under high pressure using a mixture of PbO (99.9%, Kanto Chemical), PbF_2_ (99.999%, Soekawa Chemical) and Fe_2_O_3_ (99.99%, Rare Metallic).^32^ A stoichiometric mixture of the starting materials was dried under reduced pressure at ∼573 K overnight. The mixture was sealed in an Au capsule (0.2 mm thick, 3.1 mm inner diameter and 3.2 mm depth), and the loaded capsule was subsequently inserted into a NaCl sleeve. The sleeve and capsule were inserted into a pyrophyllite cube block (one side 13 mm) with a cylindrical graphite heater. The mixture was reacted in a TRY cubic multianvil-type high-pressure apparatus (NAMO 2001) at 6.0 GPa and 1173 K for 30 min and was subsequently quenched to room temperature.

### Fabrication of PbFeO_2_F/FTO electrodes

The PbFeO_2_F electrodes were fabricated using an electrophoretic deposition method.^33^ Electrophoretic deposition was performed in a 50 mL acetone solution (>99.5%, Kanto Chemical) containing 100 mg of PbFeO_2_F powder and 20 mg of I_2_ (>99.8%, FUJIFILM Wako Pure Chemical). Two parallel fluorine-doped tin oxide (FTO) glass substrates were immersed ∼15 mm apart in the solution, and a potential of 30 V was applied for 30 s using a stabilized DC power supply (PSW 80-13.5, GW Instek). The as-fabricated electrodes were then heated at 573 K for 1 h in air in the case of electrodes not subjected to subsequent modifications.

### Ti(OCH(CH_3_)_2_)_4_ treatment and Co–Pi electrodeposition for PbFeO_2_F/FTO electrodes

According to the previously reported method,^34^ Ti(OCH(CH_3_)_2_)_4_ treatment was carried out by dipping the PbFeO_2_F/FTO electrode in an ethanol solution of 0.1 M Ti(OCH(CH_3_)_2_)_4_ (>97%, Kanto Chemical), followed by drying on a hotplate at ∼423 K. The procedure was repeated five times. Finally, the electrode was heated in air at 573 K for 1 h. Cobalt phosphate (Co–Pi) cocatalyst, known as a water-oxidation promoter,^35^ was then electrodeposited onto the TiO_2_/PbFeO_2_F/FTO electrode.^35,36^ A three-electrode cell was used with the TiO_2_/PbFeO_2_F/FTO as the working electrode, a Ag/AgCl electrode as the reference electrode and Pt wire as the counter electrode. An electrochemical bias of +1.0 V *vs.* Ag/AgCl was applied to the working electrode in 0.1 M potassium phosphate buffered at pH 7 and containing 0.5 mM cobalt nitrate (98%, FUJIFILM Wako Pure Chemical) until the charge passing through the outer circuit reached 100 mC unless otherwise stated. The pH of the phosphate solution was controlled by mixing KH_2_PO_4_ (>98.0%, Kanto Chemical), K_2_HPO_4_ (>98.0%, Kanto Chemical) and/or K_3_PO_4_ (≥98%, Sigma-Aldrich), where the concentration was maintained at 0.1 M in total.

### Characterization

A crystalline phase of the PbFeO_2_F powder was confirmed by X-ray diffraction (XRD) measurements with a Malvern Panalytical X'Pert^3^ powder diffractometer (monochromated Cu Kα). The light-absorption properties of the PbFeO_2_F powder were characterized *via* UV-vis diffuse-reflectance spectroscopy (DRS) with a JASCO V-565 spectrophotometer. Scanning electron microscopy (SEM) observations combined with energy-dispersive X-ray spectroscopy (EDS) measurements were conducted on a HITACHI S4700 equipped with an EDAX Genesis apparatus at the Materials Analysis Division, Open Facility Center, Tokyo Institute of Technology. Inductively coupled plasma optical emission spectrometry (ICP-OES) measurements were conducted with a 5100 VDV apparatus (Agilent Technologies). Measurements for Mott–Schottky plots were carried out using a BAS ALS/CHI760E electrochemical analyser. Electrochemical impedance spectroscopy measurements were performed using a potentiostat (pocketSTAT, Ivium Technologies). Mott–Schottky plots were recorded at a frequency of 100 Hz with a three-electrode-type system using the PbFeO_2_F/FTO as the working electrode, a Ag/AgCl electrode as the reference electrode (in saturated KCl aqueous solution) and Pt wire as the counter electrode in 0.1 M aqueous potassium phosphate solutions. The solutions were stirred and purged with Ar gas for 30 min before the measurements were conducted.

### Photoelectrochemical measurements

The photoelectrochemical measurements were performed with a potentiostat (HSV-110, Hokuto Denko) and an electrochemical cell with a three-electrode configuration using the as-prepared PbFeO_2_F working electrode, an Ag/AgCl reference electrode and a Pt-coil counter electrode. The cell was made of Pyrex glass. An aqueous solution of 0.1 M K_3_PO_4_ (≥98%, Sigma-Aldrich) was used as the electrolyte, which was stirred and purged with Ar gas for 30 min before the measurements were conducted. It is known that coexistence of phosphate ions in an electrolyte solution has a positive effect on electrochemical water oxidation activity of the Co–Pi catalyst,^35,37^ that and basic conditions are generally preferable for water oxidation. The light source was a 300 W Xe lamp (PE300BF, Cermax) fitted with an L42, Y48, O54, O58, R62 or R70 cutoff filter (HOYA) to emit visible light of each wavelength range. The irradiation area was 3 cm^2^. The light intensity was approximately 0.31 W cm^−2^ in the wavelength range 350–700 nm unless otherwise stated. The potentials measured against the Ag/AgCl reference (saturated KCl aqueous solution) were converted to potentials *vs.* RHE (*E*_RHE_ = *E*_Ag/AgCl_ + 0.059 pH + 0.197 at 298 K).

Incident photon to current conversion efficiency (IPCE) was measured in a similar manner using the same 300 W xenon lamp fitted with an L38 cutoff filter and a band-pass filter centred at 420 nm (HOYA). The IPCE was calculated by the following equation:IPCE (%) = 1240 × *i* (mA cm^−2^)/(*λ* (nm) × *φ* (mW cm^−2^)) × 100where *i*, *λ*, and *φ* is the photocurrent density measured under an irradiation of incident light, the incident light wavelength, and the intensity of incident photon (11 mW cm^−2^), respectively. The irradiation area was 0.28 cm^2^.

### Quantifying electrochemical O_2_ evolution

To quantify the O_2_ evolved during controlled-potential electrolysis, electrochemical measurements were performed in a gastight H-type electrochemical cell with two chambers divided by a perfluorinated membrane (Nafion 117, Sigma-Aldrich). The PbFeO_2_F/FTO working electrode and an Ag/AgCl reference electrode were separated from a Pt-wire counter electrode in each chamber. The other conditions were identical to those mentioned in the description of the photoelectrochemical measurements. The evolved O_2_ was detected using a gas chromatograph (MGC3000A, Inficon) equipped with a thermal conductivity detector and an MS-5A column. Ar gas was used as the carrier gas.

## Results and discussion

### Light absorption behaviour and flat-band potential of PbFeO_2_F

**Fig. 2:**
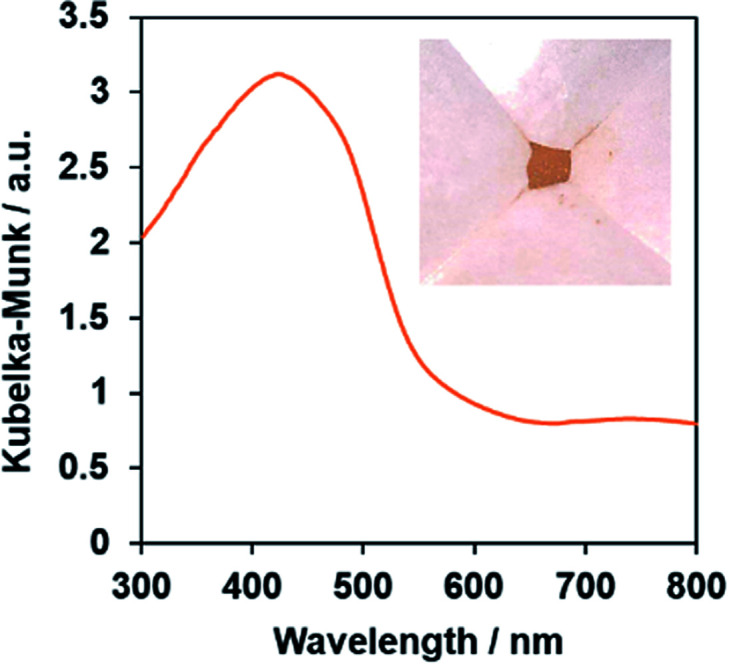
UV-vis diffuse-reflectance spectrum of the as-prepared PbFeO_2_F. The inset shows a photograph of the same material.

The single-phase production of the as-synthesized PbFeO_2_F was confirmed by XRD measurement (Fig. S1†). SEM observations show that the synthesized PbFeO_2_F consisted of 0.1–10 μm particles (Fig. S2†). The UV-vis DRS spectra of the PbFeO_2_F (Fig. 2) indicate that the material has an absorption edge at ∼600 nm and substantial absorption in the longer-wavelength region, which might be attributable to anionic defects. As reported for α-Fe_2_O_3_, the longer-wavelength absorption band is assigned to oxygen vacancies.^38,39^ The bandgap of the PbFeO_2_F was estimated to be 2.1 eV on the basis of the onset wavelength in the UV-visible DRS spectra. The previously reported PbFeO_2_F exhibited a yellow colour,^30^ whereas the as-prepared PbFeO_2_F in the present work was yellow-brown. This difference in colour originates from different concentrations of anionic defects (*i.e.*, different concentrations of reduced metal ions), which commonly affect the appearance of powders.^40^

**Fig. 3:**
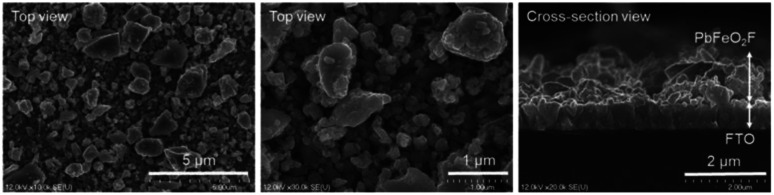
SEM images of the PbFeO_2_F/FTO electrode.

The as-synthesized PbFeO_2_F was deposited onto a FTO substrate *via* electrophoretic deposition. As shown in Fig. 3, the thickness of the deposited PbFeO_2_F particles was 1–2 μm. In the electrophoresis method, colloidal particles suspended in liquid migrate in an electric field between two electrodes, undergoing deposition onto one side of the two electrodes.^33^ Therefore, light-weight, smaller particles are preferentially deposited onto the electrode. Therefore, it is considered that the size of the deposited PbFeO_2_F particles (0.1–2 μm) were smaller than the as-synthesized PbFeO_2_F ones (0.1–10 μm).

**Fig. 4:**
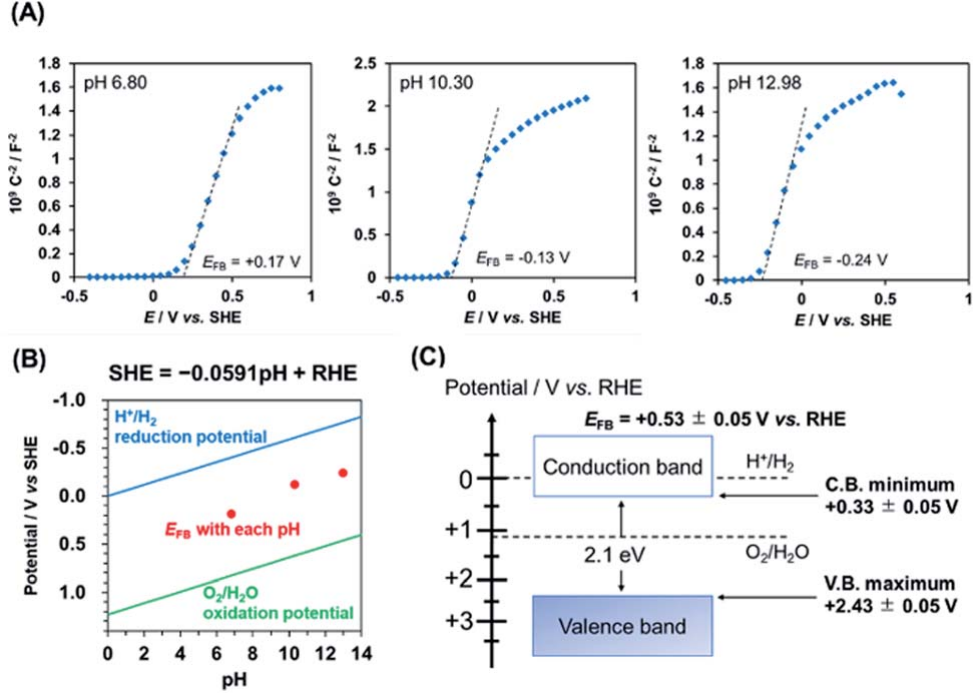
(A) Mott–Schottky plots for a PbFeO_2_F/FTO electrode recorded at 100 Hz in 0.1 M aqueous phosphate solutions with different pH values. These measurements were conducted in nonacidic solutions because of the potential dissolution of PbFeO_2_F for a strong acid. (B) pH dependence of the *E*_FB_ of PbFeO_2_F, along with water reduction/oxidation potentials. (C) Conduction and valence band-edge potentials of PbFeO_2_F, as determined from the Mott–Schottky plots and the UV-vis diffuse-reflectance spectra.

To determine the flat-band potential (*E*_FB_) of PbFeO_2_F, Mott–Schottky plots of the PbFeO_2_F/FTO electrode were recorded in aqueous phosphate solutions with different pH values under dark conditions. As shown in Fig. 4A, the Mott–Schottky plots show positive slopes irrespective of pH, which indicates n-type semiconducting behaviour of the PbFeO_2_F. The *E*_FB_ values, which were obtained by extrapolation of the linear portion to the *x*-axis intercept, were negatively shifted with increasing electrolyte pH. The negative *E*_FB_ shift corresponds to approximately −0.0591 V per pH, indicating Nernstian behaviour (Fig. 4B). Thus, the *E*_FB_ of PbFeO_2_F was determined to be +0.53 ± 0.05 V *vs.* RHE. The CBM of an n-type semiconductor depends on its conductivity and lies at 0.1–0.3 V negative relative to the *E*_FB_.^41^ Assuming that the difference between the CBM and the *E*_FB_ of PbFeO_2_F was 0.2 V because of the unclarified conductivity of PbFeO_2_F, the CBM is estimated to be +0.33 ± 0.05 V *vs.* RHE. This potential is more positive than the H^+^/H_2_ reduction potential (0 V *vs.* RHE), as displayed in Fig. 4C. On the basis of the bandgap of PbFeO_2_F (2.1 eV), the VBM of PbFeO_2_F was determined to be +2.43 ± 0.05 V, which is more positive than the water oxidation potential (+1.23 V *vs.* RHE).

### Photoelectrochemical response

**Fig. 5:**
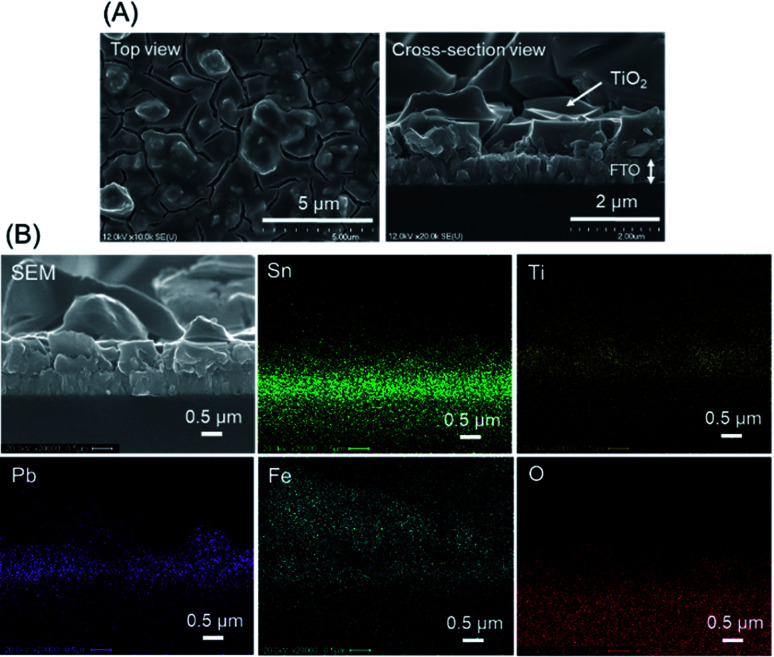
(A) SEM images of the TiO_2_/PbFeO_2_F/FTO electrode. (B) EDS mapping analysis for the TiO_2_/PbFeO_2_F/FTO electrode.

**Fig. 6:**
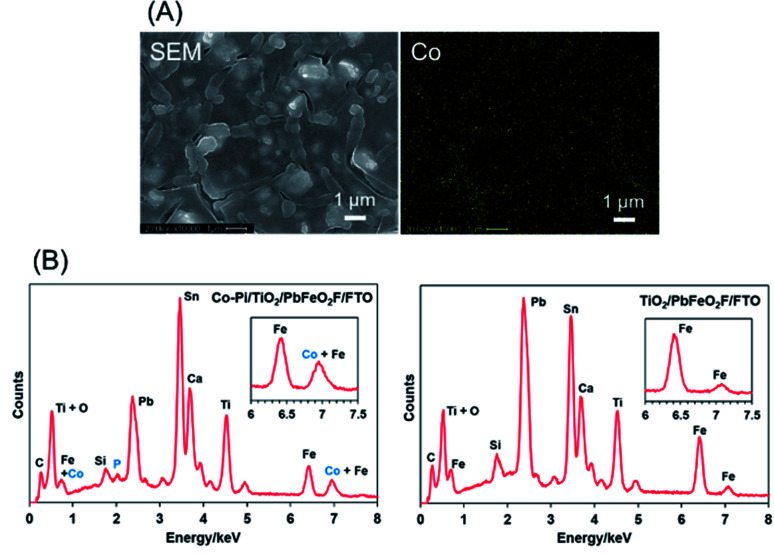
(A) EDS mapping analysis for the Co–Pi/TiO_2_/PbFeO_2_F/FTO electrode. (B) EDS spectra for (left) the Co–Pi/TiO_2_/PbFeO_2_F/FTO electrode and (right) the TiO_2_/PbFeO_2_F/FTO electrode.

The as-deposited PbFeO_2_F/FTO electrode was subjected to a post-necking treatment with an ethanol solution of titanium(iv) isopropoxide [Ti(OCH(CH_3_)_2_)_4_], followed by heating at 573 K for 1 h in air. This treatment resulted in the deposition of a TiO_2_ layer onto the PbFeO_2_F/FTO electrode (Fig. 5), which is expected to contribute to an enhanced photocurrent because of the reduced resistance of the electrode, as demonstrated in previous works.^34,42^ A Co–Pi cocatalyst, known to function as a water-oxidation promoter,^35^ was then electrodeposited onto the as-prepared TiO_2_/PbFeO_2_F/FTO electrode.^36^ The deposited Co–Pi was observed as islands on the TiO_2_/PbFeO_2_F/FTO electrode surface (Fig. 6A). EDS spectra also show the presence of P and Co species on the electrode surface (Fig. 6B). Peaks of P at 2.0 keV and Co at 6.9 keV were observed, the latter of which was overlapped with an Fe peak at 7.1 keV. In addition, EDS spot analysis demonstrated that the islands contain more P and Co species than the region surrounding the islands (Fig. S3†).

**Fig. 7:**
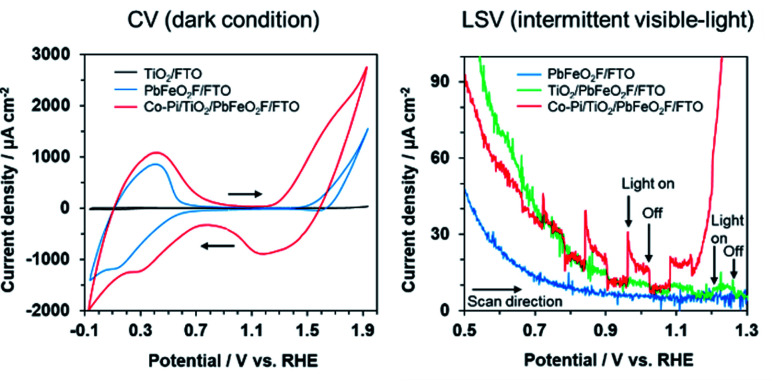
Current–potential curves for the modified PbFeO_2_F electrodes in aqueous 0.1 M K_3_PO_4_ solution (pH 12.4), as recorded at a sweep rate of (left) 100 mV s^−1^ under dark conditions and (right) 10 mV s^−1^ under intermittent visible-light irradiation. Light source: 300 W xenon lamp fitted with a Y48 cutoff filter (*λ* > 460 nm, 0.26 W cm^−2^).

Cyclic voltammetry (CV) of the modified electrodes was conducted in 0.1 M K_3_PO_4_ solution under dark conditions (Fig. 7). A TiO_2_/FTO electrode, which was prepared by Ti(OCH(CH_3_)_2_)_4_ treatment of an FTO substrate, showed little dark current in the examined potential range. By contrast, the PbFeO_2_F/FTO and Co–Pi/TiO_2_/PbFeO_2_F/FTO electrodes exhibited dark current with a redox peak in the range from 0 to +0.6 V *vs.* RHE. This dark current might be attributable to a redox reaction involving Fe cations in PbFeO_2_F because the dark current was observed when PbFeO_2_F was present. A similar CV profile has been reported for an α-Fe_2_O_3_ photoanode in the same potential range investigated in the present study.^43^ For the PbFeO_2_F/FTO electrode, a dark current with an irreversible wave in the range from +1.4 V *vs.* RHE was observed, attributable to the oxygen evolution reaction and/or the oxidation of Fe^3+^ to Fe^4+^.^43^ The oxidation of Pb^2+^ to Pb^4+^ can contribute to the dark current as well.^44^ The Co–Pi/TiO_2_/PbFeO_2_F/FTO electrode also gave a dark current with a redox peak in the range from +0.9 to +1.8 V. The dark current originated from Co species on the Co–Pi electrodeposited electrode.^35,45,46^ This interpretation is supported by an increase in the dark current for electrodes that contained more Co–Pi cocatalyst (Fig. S4†). Given these results, photoelectrochemical measurements were conducted in the potential range from +0.5 to +1.3 V to avoid the dark current during the positive sweep scan. The feasibility of using PbFeO_2_F as a water-oxidation electrocatalyst will be discussed in a later section.

Linear-sweep voltammetry of the modified electrodes in 0.1 M K_3_PO_4_ solution was conducted under intermittent visible-light irradiation (Fig. 7). The PbFeO_2_F/FTO electrode exhibited no photocurrent response. By contrast, a slight photocurrent response was observed with the TiO_2_/PbFeO_2_F/FTO electrode, primarily because of reduced interparticle resistance in the electrode.^34,47^ As displayed in Fig. S5,† electrochemical impedance spectroscopy confirmed that the charge-transfer resistance of the TiO_2_-deposited electrode was smaller than that of the electrode without TiO_2_, as indicated by the smaller arc of the semicircle in the Nyquist plots. The reduction of the charge-transfer resistance originates from improved conductivity of the electrode, which was achieved as a result of the TiO_2_ treatment.

As previously mentioned, the CBM of PbFeO_2_F was located at +0.33 ± 0.05 V *vs.* RHE (Fig. 4), which is more positive than the reported CBM of TiO_2_ (−0.04 V *vs.* RHE).^48^ Therefore, charge transfer from the CBM of PbFeO_2_F to that of TiO_2_ is apparently not efficient. However, TiO_2_ has midgap states that originate from defective sites (∼0.4 V below the CBM).^49^ In the shallow defect states, the trapped electrons can be thermally detrapped and exhibit high mobility.^50^ This effect is inferred to have improved interparticle conductivity, which is known to function as an electron trapping–detrapping effect in TiO_2_-based dye-sensitized solar cells.^51^

A clear anodic photocurrent was observed for the Co–Pi/TiO_2_/PbFeO_2_F/FTO electrode (Fig. 7). Loading the Co–Pi cocatalyst resulted in improved rate of charge transfer at the electrode interface for water oxidation and in charge separation from the surface to the bulk.^35,52^ This effect was confirmed by electrochemical impedance spectroscopy measurements (Fig. S5†). In addition, the anodic photoresponse again showed n-type semiconducting character of PbFeO_2_F, with a photocurrent onset potential of +0.7 V *vs.* RHE, although an accurate determination was difficult because of an overlap of dark current. The photocurrent onset potential of the Co–Pi/TiO_2_/PbFeO_2_F/FTO electrode, which can be regarded as the flat-band potential of PbFeO_2_F, was slightly more positive than that determined from the corresponding Mott–Schottky plot (+0.53 ± 0.05 V, Fig. 4). This result implies that charge recombination in the illuminated PbFeO_2_F surface was substantial and concealed the real flat-band potential, similar to the case of α-Fe_2_O_3_ photoanodes.^9,53^

### Photoelectrochemical activity under a wide range of visible light

**Fig. 8:**
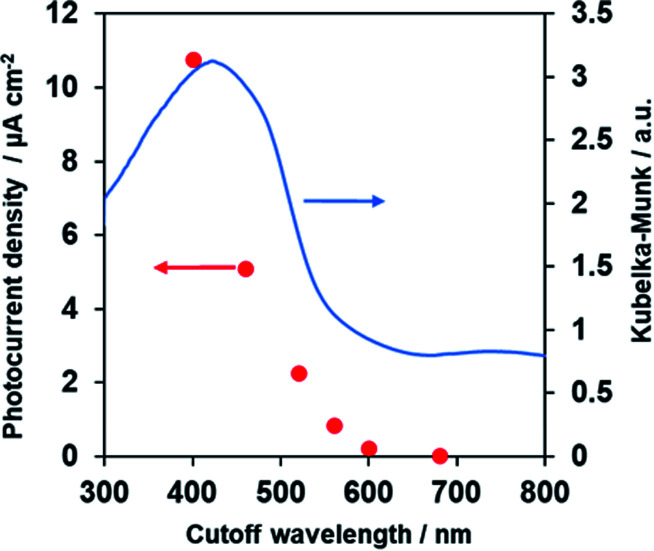
Photocurrent densities of the Co–Pi/TiO_2_/PbFeO_2_F/FTO electrode at +1.0 V *vs.* RHE in aqueous 0.1 M K_3_PO_4_ solution (pH 13.4) as a function of the cutoff wavelength of the incident light. Light source: 300 W xenon lamp fitted with cutoff filters (L42, Y48, O54, O58, R62 or R70). The diffuse reflectance spectrum of PbFeO_2_F powder is also shown.

Fig. 8 demonstrates anodic photocurrent densities of the Co–Pi/TiO_2_/PbFeO_2_F/FTO electrode at +1.0 V *vs.* RHE in aqueous 0.1 M K_3_PO_4_ solution (pH 13.4) under irradiation with light of different wavelengths, which was controlled using different cutoff filters. The UV-vis DRS spectrum of the PbFeO_2_F powder is also shown in Fig. 8. The photocurrent densities decreased with increasing cutoff wavelength and became almost zero under >600 nm irradiation. This change in the anodic photocurrent corresponded to the light-absorption properties of PbFeO_2_F, indicating that the PbFeO_2_F photoanode operated under visible-light irradiation with wavelengths as long as ∼600 nm and that the anodic photoresponse occurred by light absorption of PbFeO_2_F itself. It was also confirmed that no photocurrent was generated from Co–Pi/TiO_2_/FTO electrode (Fig. S6†).

The results also indicate that an absorption band of longer wavelengths than 600 nm does not contribute to the generation of anodic photocurrent. As previously mentioned, the absorption band at longer wavelengths originates from anionic defects in PbFeO_2_F, as reported in α-Fe_2_O_3_.^38,39^ Lifetimes of charge carriers generated at defect states in a semiconductor are generally short,^54,55^ which could be the reason for the negligible photoresponse of PbFeO_2_F under >600 nm irradiation.

The stability of the anodic photocurrent was examined *via* controlled-potential photoelectrolysis at +1.0 V *vs.* RHE (Fig. S7A†). This measurement shows that the anodic photocurrent gradually decayed with increasing reaction time. The O_2_ evolution could not be quantified because of the small current that flowed during the photoelectrolysis. Nevertheless, the fact that Co–Pi (a well-known water oxidation promoter) improved the anodic photocurrent density of the PbFeO_2_F electrode (Fig. 7) strongly suggests the oxidation of water to O_2_. As previously mentioned, the oxidation of metal cations in the PbFeO_2_F/FTO electrode was suggested;^43,44^ however, the concentration of metal cations found in the electrolyte solution by ICP-OES was negligible (below ppm level).

It has been reported that stability of a photoanode for water oxidation is influenced by various factors (*e.g.*, cocatalyst, electrolyte pH, operating potential and so on).^18,47^ It is therefore expected that optimizing these factors will improve photoelectrochemical stability of PbFeO_2_F and also photocurrent density, although it is beyond the scope of this work, which aimed at developing a new electrode material based on oxyfluorides. Nevertheless, more stable, larger photocurrent from the TiO_2_/PbFeO_2_F/FTO electrode was observed in the presence of I^−^ as a reversible electron donor upon visible light than in aqueous solution without I^−^ (Fig. S7B†). This indicates that PbFeO_2_F is essentially stable toward the photooxidation reaction.

### Electrochemical water oxidation by PbFeO_2_F in the dark

**Fig. 9:**
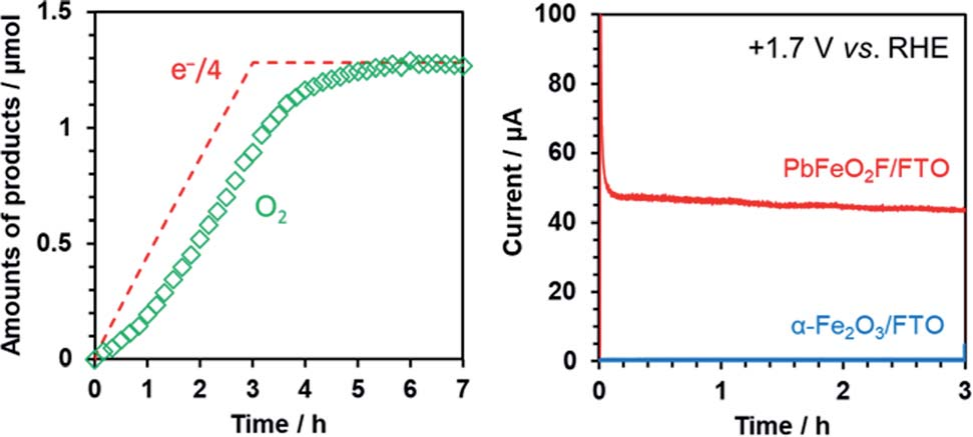
(left) Time course of O_2_ evolution during controlled-potential electrolysis at +1.7 V *vs.* RHE in aqueous 0.1 M K_3_PO_4_ solution (pH 12.9) for the PbFeO_2_F/FTO electrode under dark conditions. (right) The corresponding current–time curve for the electrode over a span of 3 h. Data for α-Fe_2_O_3_ is also shown for comparison.

The ability of PbFeO_2_F to function as an electrocatalyst for water oxidation was investigated by controlled-potential electrolysis using the as-prepared PbFeO_2_F/FTO electrode at +1.7 V *vs.* RHE under dark conditions (Fig. 9). Although the onset potential of water oxidation current by the PbFeO_2_F/FTO electrode was more negative than +1.7 V (see Fig. 7), we conducted the electrolysis experiment at +1.7 V in order to obtain more O_2_ gas for reliable quantification. GC analysis of the evolved O_2_ gas shows that the PbFeO_2_F functions as an electrocatalyst to stably produce O_2_. The amount of O_2_ evolved at the initial stage of the electrolysis was slightly smaller than one-fourth the amount of electrons that flowed to the outer circuit. This result is attributed primarily to a time lag of gas diffusion from the solution to the gas chromatograph. In fact, the total O_2_ evolved reached the value expected on the basis of the reaction stoichiometry, giving a high faradaic efficiency of 97%. This result indicates that water oxidation was the major path at the PbFeO_2_F/FTO electrode. Under the same condition, an α-Fe_2_O_3_/FTO electrode, prepared in a similar manner, did not produce appreciable current, indicative of its large overpotential for water oxidation.

## Conclusions

PbFeO_2_F synthesized by a high-pressure method had grain sizes ranging from 0.1 to 10 μm and an estimated bandgap of 2.1 eV. The Mott–Schottky plot measurements showed n-type semiconductivity of PbFeO_2_F with a flat-band potential of +0.53 ± 0.05 V *vs.* RHE. The PbFeO_2_F electrode modified with a conductive TiO_2_ layer and a Co–Pi water-oxidation cocatalyst exhibited a clear anodic photocurrent in aqueous K_3_PO_4_ solution under visible-light irradiation (*λ* < 600 nm).

At present, the performance of the PbFeO_2_F photoanode is not satisfactory; IPCE at 420 nm was 0.14% at +1.0 V *vs.* RHE. Nevertheless, it is expected that photoelectrochemical performance of PbFeO_2_F will be improved by further development in materials synthesis and post modification technologies for PbFeO_2_F, as we can learn from the history of the α-Fe_2_O_3_ photoanode.^9–11^

Meanwhile, a PbFeO_2_F/FTO electrode without the modifications exhibited anodic current and O_2_ evolution in aqueous K_3_PO_4_ solution at +1.7 V *vs.* RHE, where water oxidation did not proceed for an α-Fe_2_O_3_ electrode. The present study reveals that PbFeO_2_F becomes a bifunctional material—that is, a photoanode material that can function under a wide range of visible-light wavelengths and as an electrocatalyst at a relatively low overpotential for water oxidation.

## Author contributions

R. M. performed most of the experiments and wrote the manuscript with K. M. K. I. and Y. I. synthesized the PbFeO_2_F powder. K. M. supervised the project.

## Conflicts of interest

There are no conflicts to declare.

## Supplementary Material

RA-011-D1RA04793K-s001
