# Supplementary material for: A bifunctional lead–iron oxyfluoride, PbFeO2F, that functions as a visible-light-responsive photoanode and an electrocatalyst for water oxidation
Source: RSC Adv. 2021 Jul 23;11(41):25616–23. doi: 10.1039/d1ra04793k (PMC9037018; doi:10.1039/d1ra04793k)
Supplement: RA-011-D1RA04793K-s001 [file RA-011-D1RA04793K-s001.pdf]

Electronic Supplementary Material (ESI) for RSC Advances.
This journal is © The Royal Society of Chemistry 2021

Electronic Supplementary Information (ESI)

A bifunctional lead–iron oxyfluoride, $PbFeO_2F$, that functions as a visible-light-responsive photoanode and an electrocatalyst for water oxidation

Ryusuke Mizuochi,[1] *Kazunari Izumi,*[2] *Yoshiyuki Inaguma,*[2] *and Kazuhiko Maeda*[1,]*

[1] Department of Chemistry, School of Science, Tokyo Institute of Technology, 2-12-1-NE-2 Ookayama, Meguro-ku, Tokyo 152-8550, Japan

[2] Department of Chemistry, Faculty of Science, Gakushuin University ,1-5-1 Mejiro, Toshima-ku, Tokyo 171-8588, Japan

*To whom correspondence should be addressed.

maedak@chem.titech.ac.jp

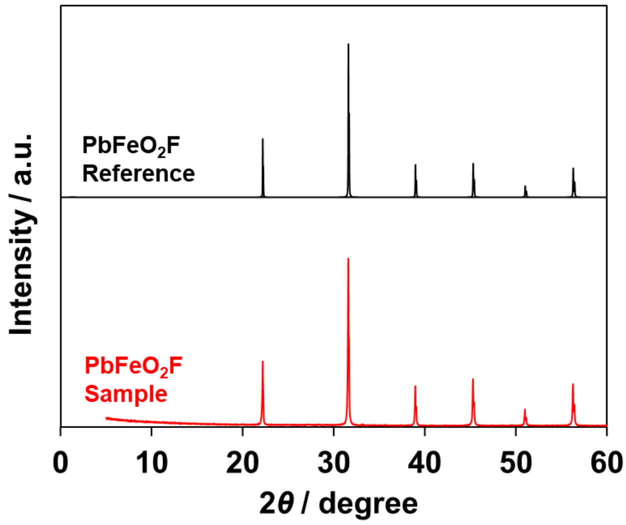

Fig. S1. XRD patterns of the synthesized $PbFeO_2F$ and the reference. The pattern of the reference was drawn using a CIF file available from the literature.[1]

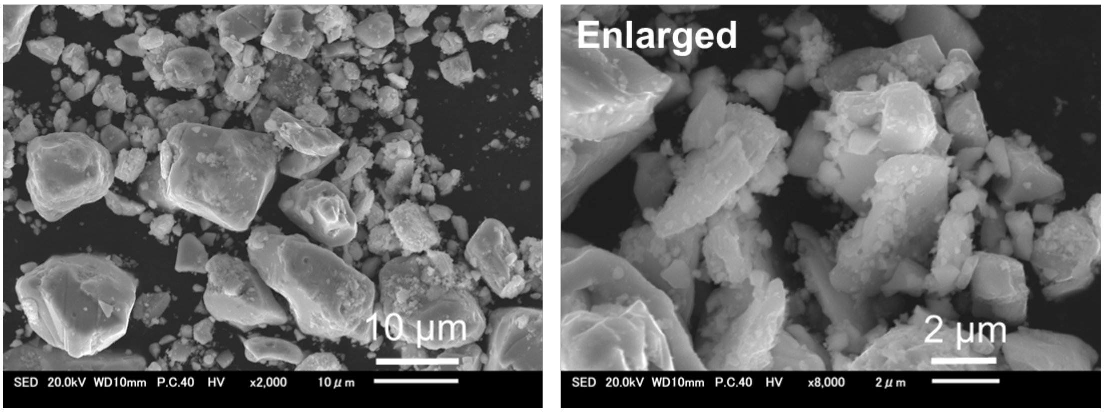

Fig. S2. SEM images of the synthesized $PbFeO_2F$ powder.

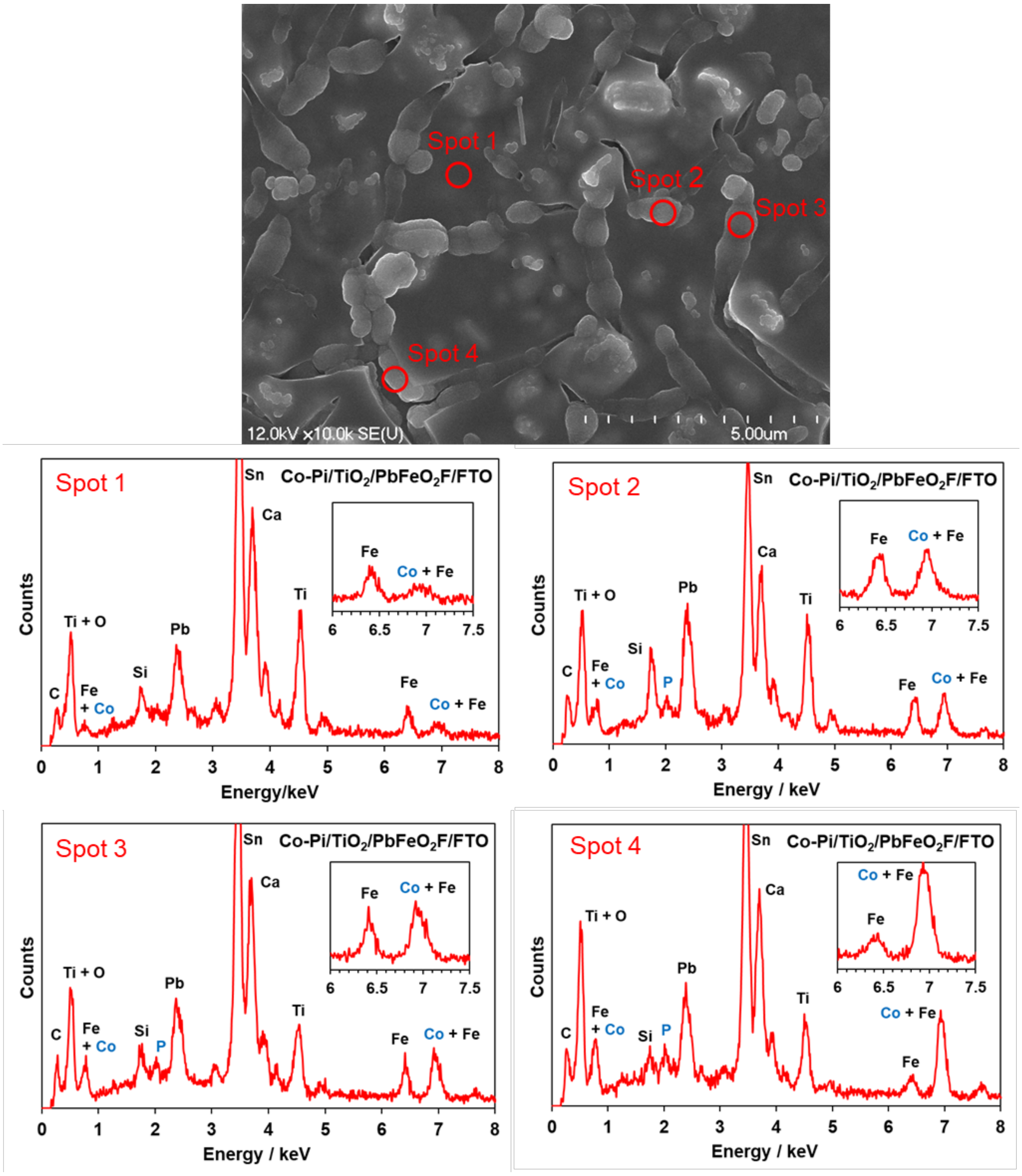

Fig. S3. EDS spectra for the Co-Pi/TiO_2/$PbFeO_2F$/FTO electrode. Spot 1 in the flat area shows a weak signal of Co at 6.9 keV. By contrast, spots 2, 3 and 4 in "islands" show an obvious contribution of P with an energy at ~2.0 keV as well as a stronger Co signal.

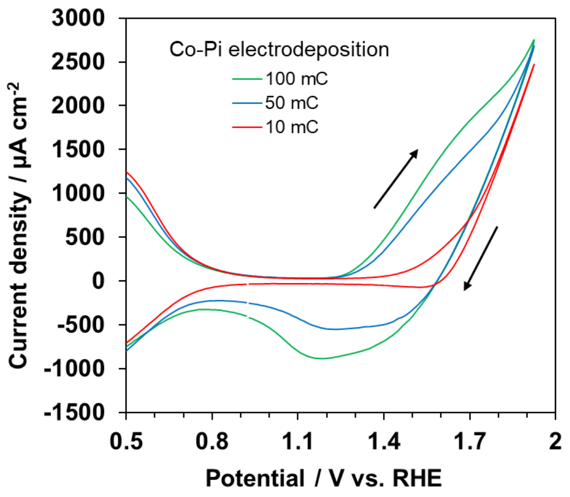

Fig. S4. Current-potential curves in aqueous 0.1 M K_3PO_4 solution (pH 12.4) for Co-Pi/TiO_2/$PbFeO_2F$/FTO electrodes. Scan rate: 100 mV s^{-1}. Co-Pi was loaded by applying +1.0 V *vs* Ag/AgCl for the TiO_2/$PbFeO_2F$/FTO electrodes in 0.1 M potassium phosphate buffered solution containing 0.5 mM cobalt nitrate (pH 7). The total charge that flowed was varied from 10 to 100 mC.

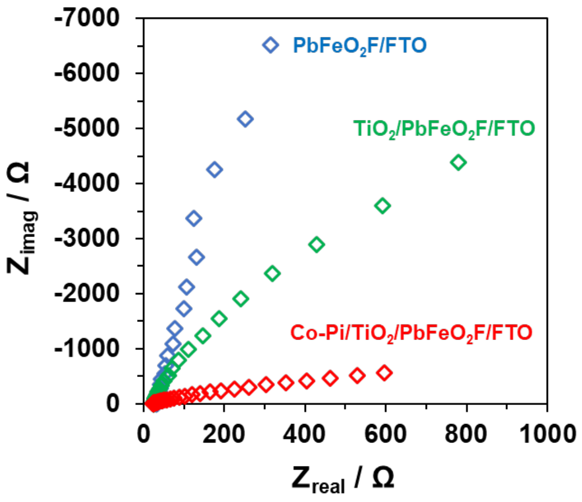

Fig. S5. Nyquist plots for the modified $PbFeO_2F$/FTO electrodes (3 cm^2) at +1.0 V *vs* RHE in aqueous 0.1 M K_3PO_4 solution (pH 12.4) under visible-light irradiation. Light source: 300 W xenon lamp fitted with an L42 cutoff filter (λ > 400 nm). The amplitude of the modulation potential was 10 mV. The frequency of the modulation potential was lowered from 10 kHz to 1 Hz.

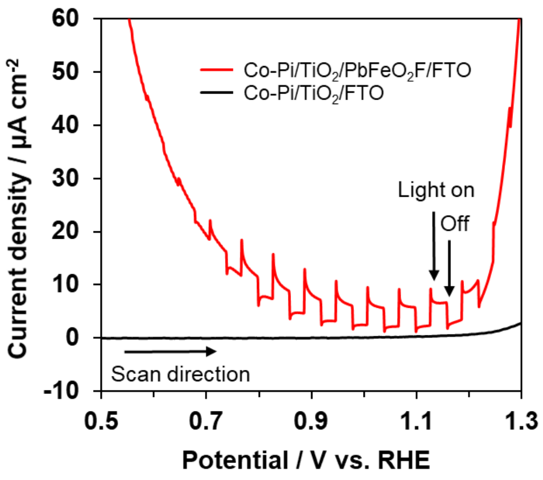

Fig. S6. Current–potential curves for the Co-Pi/TiO_2/$PbFeO_2F$/FTO and Co-Pi/TiO_2/FTO electrodes in aqueous 0.1 M K_3PO_4 solution (pH 12.4), as recorded at a sweep rate of 10 mV s^{-1} under intermittent visible-light irradiation. Light source: 300 W xenon lamp fitted with a Y48 cutoff filter ($\lambda > 460$ nm, 0.26 W cm^{-2}).

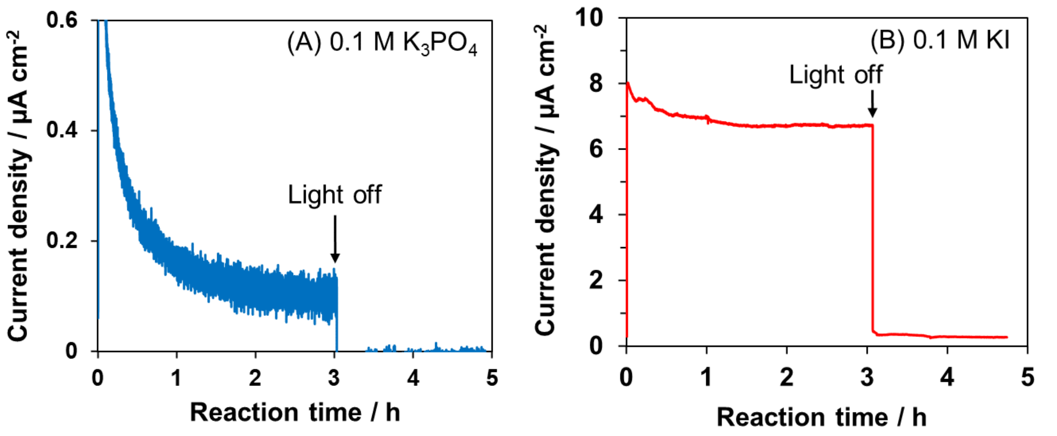

Fig. S7. Current–time curves in controlled-potential photoelectrolysis at +1.0 V *vs* RHE under visible-light irradiation. The experiments were conducted (A) in 0.1 M K_3PO_4 solution (pH 12.9) using the Co-Pi/TiO_2/$PbFeO_2F$/FTO electrode and (B) in 0.1 M KI solution (pH 6.3) using the TiO_2/$PbFeO_2F$/FTO electrode, respectively. Light source: 300 W xenon lamp fitted with an L42 cutoff filter ($\lambda > 400$ nm).

References

S1. Y. Inaguma, J.-M. Greneche, M.-P. Crosnier-Lopez, T. Katsumata, Y. Calage and J.-L. Fourquet, Structure and Mössbauer Studies of F−O Ordering in Antiferromagnetic Perovskite $PbFeO_2F$, *Chem. Mater.*, 2005, **17**, 1386-1390.
